# The clinical effects of Gyejibongnyeong-Hwan (Gui Zhi Fu Ling Wan) on patients with hyperlipidemia: A study protocol for a multicenter, double-blind, two-armed parallel, investigator-initiated, exploratory randomized controlled trial

**DOI:** 10.1097/MD.0000000000033093

**Published:** 2023-04-21

**Authors:** Mi Mi Ko, Pyung-Wha Kim, So Young Jung, Cheol-Hyun Kim, Byung-Cheol Lee, Jeeyoun Jung

**Affiliations:** a KM Science Research Division, Korea Institute of Oriental Medicine, Yuseong-daero 1672, Yuseong-gu, Daejeon, Republic of Korea; b Clinical Research Coordinating Team, Korea Institute of Oriental Medicine, Daejeon, Republic of Korea; c Department of Internal Medicine and Neuroscience, College of Korean Medicine, Wonkwang University, Iksan, Republic of Korea; d Department of Internal Medicine, College of Korean Medicine, Kyung Hee University, Seoul, Republic of Korea.

**Keywords:** dyslipidemia, Gyejibokryeong-Hwan, herbal medicine, hyperlipidemia, Korean medicine, Randomized controlled trial

## Abstract

Hyperlipidemia, the most common form of dyslipidemia, is associated with an increased risk of atherosclerotic cardiovascular diseases. There is a constant demand for therapeutic agents with relatively few side effects that can be administered from the initial stage of hyperlipidemia, herbal medicines derived from natural products can be considered candidates for treating dyslipidemia. This study aims to explore the feasibility, preliminary effectiveness, and safety of Gyejibongnyeong-Hwan (GBH) in patients with hyperlipidemia.

**Methods:**

This was a 2-armed, parallel, multicenter, and exploratory randomized controlled trial on dyslipidemia. We will recruit 90 patients aged 20 to 65 years with hyperlipidemia between November 2021 and December 2022. Eligible participants will be randomly assigned to receive GBH or placebo granules for 8 weeks and followed up for 4 weeks after 4 weeks of lifestyle modification. The primary outcome is the percentage changes in low-density lipoprotein cholesterol from baseline to week 8. The secondary outcomes are percentage changes in other blood lipid parameters, blood glucose parameters, and blood stasis scores. As an exploratory outcome measure, metabolite analysis will be conducted to observe changes in metabolic patterns.

**Discussion:**

This is the first randomized controlled trial to explore the clinical effect and safety of GBH compared to placebo control in patients with hyperlipidemia, thereby potentially facilitating better management of hyperlipidemia. The results of this pilot study could form a foundation for future large-scale confirmatory clinical trials.

**Ethics and dissemination:**

This study was permitted by the Ministry of Food and Drug Safety on investigational new drug application on August 12, 2021 and approved by the Institutional Review Board of Kyung Hee University, Seoul, Republic of Korea (KOMCIRB202110012001) on November 26, 2021. The results will be published in a peer-reviewed journal and disseminated electronically and in print.

## 1. Introduction

Hyperlipidemia, the most common form of dyslipidemia, involves abnormally elevated levels of lipids in the blood, including cholesterol and triglycerides (TG), and is associated with an increased risk of atherosclerotic cardiovascular (CV) disease.^[1]^

Recently, hyperlipidemia prevalence has been increasing worldwide over the past decades due to factors such as diet, lifestyle, and aging. The prevalence of dyslipidemia in South Korea reached over 38% in 2018, comparable to the prevalence in other countries, and the substantial disease burden has also been increasing consequently.^[2,3]^ Generally, hyperlipidemia has no clinically obvious physical signs or symptoms; it is usually detected through blood tests.

nonpharmacological interventions, such as dietary, exercise, and other lifestyle changes, are recommended for the primary prevention of hyperlipidemia before considering medication. However, if appropriate medication is required in high-risk patients, statins (3-hydroxy-3-methyl-glutaryl-coenzyme reductase inhibitor) are considered first-line agents.^[2]^ Particularly, low-density lipoprotein cholesterol (LDL-C) is a well-known risk factor for coronary artery disease and a major lipid indicator that determines the risk assessment and treatment principle of CV disease.^[4]^ In actual clinical and treatment guidelines, the primary treatment goal for dyslipidemia is to set the LDL-C target level according to the presence or absence of CV disease and its risk, and statin treatment is widely prescribed as the primary treatment for lowering LDL-C levels. However, significant residual CV risk has been noted in several trials of statin therapy, and consequently, there has been increased interest in modulating nonLDL-C levels, such as TG and HDL-C levels, to achieve further reduction in residual CV risk during statin treatment.^[5]^ In addition, in the case of statin drugs, side effects such as digestive disorders, heartburn, liver toxicity, rhabdomyolysis, and diabetes remain a concern.^[6–8]^ Therefore, there is a constant demand for a therapeutic agent with relatively good efficiency and few side effects that can be administered from the initial stage of hyperlipidemia. Herbal medicines derived from natural products can be considered an option for treating dyslipidemia.

In traditional East Asian medicine, hyperlipidemia is regarded as the retention of phlegm and fluid disease, having similar etiology and pathogenesis based on spleen-deficiency and phlegm-stagnation, accumulation and stasis of heat, and qi and blood stagnation induced by phlegm-damp, water-dampness, and blood stasis. Therefore, strengthening the spleen, dissolving phlegm, clearing away heat and diuresis, supplementing qi, and activating blood circulation are commonly used therapeutic methods for hyperlipidemia.^[9–13]^

In Korea, (Gyejibongnyeong-Hwan [GBH]; Gui Zhi Fu Ling Wan in traditional Chinese; and Keishibukuryogan in Kampo medicine) is an herbal medicine comprising 5 herbs, including *Cinnamomum cassia* Blume, *Poria cocos* Wolf, *Paeonia suffruticosa* Andrews, *Prunus persica* Batsch, and *Paeonia lactiflora* Pallas, widely used to treat several symptoms of blood stasis, which collectively refers to pathological conditions of blood circulation disorder in clinics.^[14,15]^ It has been reported to have antilipid, antiplatelet, antithrombotic, and antiatherosclerosis.^[14]^ In particular, a significant number of preclinical and clinical studies have been reported that have identified the blood lipid-lowering effects of GBH.^[14]^

However, few clinical studies have evaluated the effects of GBH in patients with dyslipidemia in Korea. In other studies, the target lipid parameters of GBH, such as total cholesterol (TC), TG, and LDL-C, have been reported differently according to clinical studies.^[16–21]^

Therefore, this study aims to evaluate the clinical effect and safety of GBH on serum lipid levels and CV risk in patients with borderline hyperlipidemia.

## 2. Methods

### 2.1. Study design and setting

**Figure 1.**
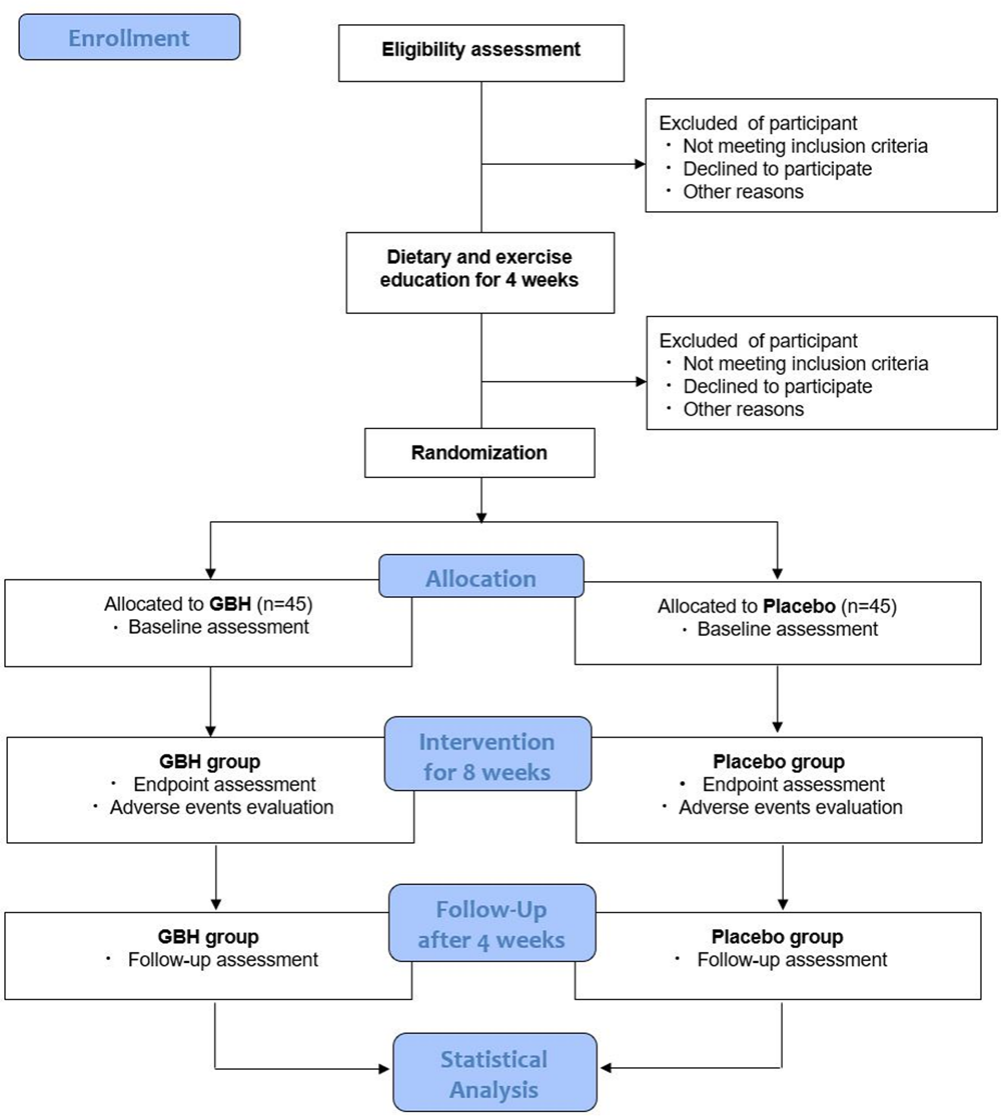
Study flow chart. GBH, Gyejibongnyeong-Hwan.

This is a study protocol for multi-center, randomized, double-blind, placebo-controlled, parallel, investigator-initiated pilot study. Subjects will be recruited from 2 hospitals, Kyung Hee University Oriental Medical Center (Seoul) and Wonkwang University, Gwangju Medical Center (Gwangju), between November 2021 and December 2022. The study period will include 4 weeks of lifestyle modification, 8 weeks of medication, and 4 weeks of follow-up. The study period procedure is summarized in the Consolidated Standards of Reporting Trials (CONSORT) diagram (Fig. 1), and the schedule of enrollment, intervention, and assessments is summarized in Table 1. The trial will be performed in accordance with the Declaration of Helsinki and the Good Clinical Practice Guidelines. This study was sponsored and managed by the Korea Institute of Oriental Medicine (KIOM) during the study period. KIOM wrote the protocol following the CONSORT Extension for Chinese Herbal Medicine Formulas 2017^[22]^ and Standard Protocol Items: Recommendations for Interventional Trials 2013 statement.^[23]^

**Table 1:** Schedule of enrollment, interventions, and assessments.

	Study period					
	Screening		Post allocation			Follow-up
Time point	1st (−4 w)	2nd (±7 d)	Baseline (−14 d)	W 4 (±7 d)	W 8 (±7 d)	W 12 (±7 d)
Enrollment						
Informed consent	●					
Eligibility screen	●					
Demographic characteristics	●					
Medical history	●					
Random allocation			●			
Interventions						
Dietary and exercise education	●	●	●	●	●	
GBH or placebo			← - - - - - - - - - - - - - - - - - - - - →			
Assessments						
Vital sign	●	●	●	●	●	●
Laboratory testing*	●	●		●	●	●
Pregnancy testing		●		●		
Blood stasis questionnaire	●		●		●	
Blinding test					●	
Adverse events			●	●	●	●
Medication compliance				●	●	

GBH = Gyejibongnyeong-Hwan.

* Including hematological test, blood lipid parameters, blood glucose parameter, liver and renal function test, and urine test.

### 2.2. Study registration

This study is registered with the Clinical Research Information Service (https://cris.nih.go.kr/cris/en/) (KCT0006842). The current protocol versions are 1.1.

### 2.3. Eligibility criteria

#### 2.3.1. Inclusion criteria.

1. Adults aged 19 years or over but under 65 years.
2. Eligible subjects through screening tests and whose lipid profile results after 4 weeks of lifestyle modification correspond to 1 or more of the following: (a) TC ≥ 200 mg/dL, (b) TG 150 to 499 mg/dL, (c) LDL-cholesterol 130 to 250 mg/dL, and (d) HDL-cholesterol < 40 mg/dL (However, even if the subject corresponds to more than 1, LDL-cholesterol should not exceed 250 mg/dL, and TG should not exceed 499 mg/dL).
3. Voluntary signed written informed consent after sufficient explanation of this study.

#### 2.3.2. Exclusion criteria.

1. Subjects with a history of hypersensitivity or allergies to the study medication components.
2. Subjects who have a history of unstable angina, myocardial infarction, atherosclerotic ischemic stroke, transient ischemic attack, cerebrovascular disease, coronary artery disease, coronary artery bypass graft, percutaneous coronary intervention, peripheral arterial disease, carotid artery disease (significant carotid artery stenosis), or abdominal aneurysm; any malignant tumor within 5 years before screening; drug or alcohol abuse; gastrointestinal surgery or drug absorption disorders due to gastrointestinal disorders.
3. Subjects with unstable hypertension (systolic blood pressure ≥ 180 mm Hg or diastolic blood pressure, diastolic blood pressure ≥ 110 mm Hg), diabetes (taking diabetes medication or fasting blood glucose > 160 mg/dL), aspartate transaminase or alanine transaminase  ≥ 2 × upper normal limit (UNL), estimated glomerular filtration rate  < 30mL/min or creatinine ≥ 2 × UNL; creatine kinase > 2 × UNL or a history of myopathy or rhabdomyolysis, thyroidal disease, pancreatitis.
4. Subjects who have administered a concomitant drug during the clinical trial or are judged to have unavoidable administration of a concomitant drug.
5. Subjects who used drugs and food that can affect lipid profile within 4 weeks prior to the screening (statins, fibrates, bile acid sequestrants, niacin, anti-obesity drugs, steroids for systemic action, fish oil, omega-3, cholestin products, fiber-based laxatives, and phytosterol margarine, among others).
6. Subjects who participated in other clinical trials within the last 3 months.
7. Female subjects who are pregnant or lactating.
8. Female subjects who plan to conceive.
9. Subjects who the investigator considers to be inappropriate to participate in the trial.

### 2.4. Recruitment

The trial will be conducted at 2 hospitals as follows: Kyung Hee University Oriental Medical Center and Wonkwang University Gwangju Medical Center. Posters will be posted publicly, both inside and outside the hospitals. Investigators will provide research information, including the objectives, procedures, and potential benefits and risks, to participants through standardized interviews before their participation, and informed consent will be obtained from all participants. Participants will be allowed to withdraw from the study at any time without disadvantage and will be immediately notified when new information regarding the study is obtained.

### 2.5. Interventions

Before allocation into groups, all participants will be educated and instructed to follow dietary and exercise guidelines for hyperlipidemia using a brochure based on the Guidelines by the Korean Ministry of Food and Drug Safety for clinical trials for hyperlipidemia treatment for 4 weeks.^[24]^ After 4 weeks of lifestyle modification, eligible participants will be randomly assigned to receive GBH or placebo granules for 8 weeks. GBH, a light brown granular substance, will be prepared by Hanpoong Pharm and Foods Co., Ltd. (Batch number: 20001; Wanju, Jeollabuk-do, South Korea). It will be produced according to the Korean Good Manufacturing Practice guidelines outlined by Korea Ministry of Food and Drug Safety and manufactured so that there is no difference between them in shape, color, smell, or taste. A blinded pharmacist will check the storage, dispensation, and quality of the investigational products. GBH is made of an extract comprising the following herbs: *Cinnamomum cassia* Blume (1.33 g), *Poria cocos* Wolf (1.33 g), *Paeonia suffruticosa* Andrews (1.33 g), *Prunus persica* Batsch, and *Paeonia lactiflora Pallas* (1.33 g), which were extracted with 8 to 10 times the volume of water at 80°C to 100°C for 2 to 3 hours. The extract was filtered, and the filtrate was concentrated under reduced pressure at 60°C or lower to obtain the herbal extract. The placebo granule was made of corn starch, lactose hydrate, hydroxypropyl methylcellulose, Opadry® 200, and caramel coloring.

Participants will be instructed to take 1 pack (4.0 g) 3 times daily for 8 weeks. The dosage regimen is based on an authorization from the Korean Ministry of Food and Drug Safety. Participants will be instructed to return unopened packages of the investigational product to check their adherence to the treatment. If any adverse events occur, investigators will take appropriate action depending on the severity of the adverse event and its causal relationship with the investigational product.

Co-administration of medications used for managing metabolic diseases, such as hypertension and diabetes, and dietary supplements that do not directly affect hyperlipidemia can be permitted during the study period. However, participants will be instructed not to change the dose or type of concomitant medication as much as possible, if they are changed, and the investigators will record the details in the case report form (CRF).

The use of fibrates, bile acid sequestrants, niacin, anti-obesity drugs, steroids intended for systemic action, fish oil, colestine products, fiber-based laxatives, phytosterol margarine, which can affect the lipid profile, the drugs usage that can affect thyroid function, such as thyroxine, and will be prohibited during the study period.

### 2.6. Outcomes measures

#### 2.6.1. Primary outcome.

1. Percentage changes in LDL-C from the baseline to week 8.

#### 2.6.2. Secondary outcomes.

1. Percentage changes in TC, HDL-C, TG, apolipoprotein A1, and apolipoprotein B from the baseline to week 8.
2. Other blood lipid parameters.
3. Changes in the total scores of blood stasis questionnaire.

#### 2.6.3. Exploratory outcomes.

As an exploratory outcome measure, metabolite analysis will be conducted to observe the changes in metabolic patterns.

### 2.7. Safety and adverse events

The safety of the investigational product will be measured by comparing the laboratory liver and renal function test results (aspartate transaminase, alanine transaminase, gamma- glutamyl transferase, blood urea nitrogen, and creatinine) before and after the intervention in each group. Investigators will also examine the adverse events at each visit, which will be recorded in this study. Additionally, and investigators will ensure that all participants with adverse reactions receive adequate medical attention and follow-up until symptoms are resolved.

### 2.8. Sample size calculation

This is a preliminary exploratory study to assess the clinical effect and safety of GBH on serum lipid levels and CV risk in patients with borderline hyperlipidemia. According to a study that recommended the minimum sample size for the preliminary study,^[25]^ the minimum effect size was assumed to be 0.20, a dropout rate of 20% was considered, and a total of 90 participants (45 in each group) are set as the target sample size.

### 2.9. Randomization and allocation concealment

An independent statistician will generate a random assignment code using the block randomization method with block sizes in SAS® Version 9.4 (SAS Institute Inc., Cary, NC). Participants who satisfy the eligibility criteria will be assigned to the GBH or placebo granule group at the baseline visit (week 0) with a 1:1 allocation ratio. The generated code will be sealed in opaque envelopes and stored in double-locked cabinets. We will maintain allocation concealment throughout the study period by providing participants with identically packaged, consecutively numbered drug containers.

### 2.10. Blinding

The participants, investigators, outcome assessors, pharmacists, study monitors, and data managers will be blinded to which medication will be administered to participants using placebo granules as a control. An independent statistician who generated the random assignment code will provide the random assignment table directly to Hanpoong Pharm & Foods Co., Ltd. (Jeonju, Republic of Korea), and the company will pack and deliver investigational products to the hospital. The placebo granules will have the same formulation and properties as the HHT granules to prevent any bias in the efficacy and safety assessments. Blinding will be maintained until all participants have completed the study unless serious medical emergencies occur to them. When unblinding is necessary, such as in severe medical emergencies, investigators will open a separate emergency code stored in double-locked cabinets. Investigators must document the reasons for the unblinding. To check whether participant blinding is maintained during the study period, investigators will perform a blinding test on participants after 8 weeks of investigational product administration (Visit 5). Investigators will ask the participants which type of drug they think was administrated: “real drug,” “false drug,” or “do not know.” The success of blinding will be evaluated using the new blinding index and a 95% confidence interval.^[26]^

### 2.11. Data and safety monitoring

We will use a web based electronic CRF for data collection and verification. This will be managed by the data management team from the KIOM. The electronic CRF is accessible only to investigators who are directly involved in the clinical trial and receive relevant education. The range of the data values will be set in advance to ensure the data quality. Although data entry will be performed only once by the investigators, data quality will be managed by 2 verification processes for each clinical research associate and data manager. A clinical research associate at KIOM, a supporting institution, will visit the hospital regularly to monitor protocol violations, recruitment rates, document reporting, and adverse events (including severe adverse events) during the study period. The detected items will be adequately resolved through discussions with the investigators. No formal data-monitoring committee will be convened. Currently, there is no planned audit. However, auditing can be conducted to preserve the integrity of the trial by a quality assurance team at the supporting institution, independent of the investigators.

### 2.12. Statistical analysis

An independent statistician will conduct statistical analysis using SAS® Version 9.4 (SAS Institute Inc., Cary, NC). According to principle, an efficacy analysis will be conducted using a full analysis set according to the intention-to-treatment principle, including all randomly assigned participants who received at least 1 evaluation after the investigational product has been prescribed at least once. If necessary, per-protocol set analysis will also be conducted, including only participants who have completed the entire process as described in the protocol and have no significant violations. Participants with < 70% compliance with the investigational product will be excluded from the per-protocol set analysis. The safety analysis will include all data obtained from participants who have taken at least 1 investigational product.

The demographic and general clinical characteristics of the participants will be summarized by grouping. Continuous data will be presented as mean and 95% confidence intervals or medians according to the data distribution, and dichotomous data as frequency and percentage. Continuous data will be analyzed using the independent t-test or Wilcoxon rank-sum test, and dichotomous data will be analyzed using the chi-square test or Fisher exact test.

The primary outcome of this study is the mean change in LDL-C levels from baseline to week 8. A 2-sided test with a significance level of 5% will be performed using an analysis of covariance with baseline as the covariate and the treatment group as the fixed factors. The intragroup changes in outcome measures from baseline to posttreatment will be analyzed using the paired *t* test or Wilcoxon signed-rank test for continuous variables and the chi-square test or Fisher exact test for dichotomous variables. Furthermore, repeated-measures analysis of variance will be used to compare the differences in trends between visits in each group. Missing values will be replaced with the multiple imputation method. If necessary, subgroup analysis can be performed by categorizing the initial characteristics of participants at the screening or baseline visit.

## 3. Ethics and dissemination

This trial will be performed in accordance with the Declaration of Helsinki and the Good Clinical Practice Guidelines and CONSORT Guidelines. This study was permitted by the ministry of food and drug safety on investigational new drug application on August 12, 2021 and approved by the IRB of Kyung Hee University, Seoul, Republic of Korea (KOMCIRB202110012001) on November 26, 2021. Written informed consent will be obtained from all participants prior to their enrollment in the study. The results will be published in a peer-reviewed journal and disseminated electronically and in print, regardless of the results.

## 4. Discussion

This is a study protocol for a randomized, double-blind, placebo-controlled, parallel, investigator-initiated pilot clinical trial evaluating the effect of GBH on patients with borderline hyperlipidemia. We will evaluate the preliminary efficacy and safety of an 8-week administration of GBH compared with placebo granules and assess the feasibility of large-scale randomized controlled trials (RCTs). To the best of our knowledge, this is the first RCT to assess the effect of GBH in patients with hyperlipidemia.

GBH is 1 of the most frequently prescribed herbal medicines for patients with impaired blood circulation, known as blood stasis syndrome in Korea, China and Japan.^[27]^ The main components of GBH, such as amygdalin derived from *Persicae Semen*, paeoniflorin derived from *Paeoniae Radix*, and cinnamic acid derived from *Cinnamomi ramulas*, have antilipid, antiplatelet, antithrombosis, and antiarteriosclerosis effects.^[14]^

GBH is not approved by the ministry of food and drug safety as a medicine for dyslipidemia^[15]^ but a significant number of preclinical and clinical studies have been reported that have identified the blood lipid-lowering effects of GBH.^[16–21]^

If the treatment effect and safety of GBH compared to placebo granules are found to be promising in this study and the study design is feasible, we will design a confirmatory, large-scale RCT based on the results of the study to draw definitive conclusions regarding the efficacy of GBH.

In conclusion, this article presents a protocol for a rigorously well-designed, double-blind, placebo-controlled pilot RCT of GBH in patients with hyperlipidemia. The strengths of our study include using placebo granules as the control and using the objective blood lipid parameter, LDL-C, as the primary outcome to minimize performance bias and detection bias. The findings of this study are expected to provide a basis for large-scale, confirmatory RCT to confirm the efficacy and safety of GBH in treating patients with hyperlipidemia. Additionally, stronger evidence for GBH as a treatment option for hyperlipidemia may be provided to clinicians, patients, and researchers.

## Author contributions

**Conceptualization:** Mi Mi Ko, Pyung-Wha Kim, Cheol-Hyun Kim, Byung-Cheol Lee, Jeeyoun Jung.

**Methodology:** Mi Mi Ko, Pyung-Wha Kim, Jeeyoun Jung.

**Resources:** Mi Mi Ko, Pyung-Wha Kim, So Young Jung, Cheol-Hyun Kim, Byung-Cheol Lee, Jeeyoun Jung.

**Supervision:** Jeeyoun Jung.

**Writing – original draft:** Mi Mi Ko, Pyung-Wha Kim.

**Writing – review & editing:** Mi Mi Ko, Pyung-Wha Kim, Cheol-Hyun Kim, Byung-Cheol Lee, Jeeyoun Jung.
